# Point Cloud Scene Completion of Obstructed Building Facades with Generative Adversarial Inpainting

**DOI:** 10.3390/s20185029

**Published:** 2020-09-04

**Authors:** Jingdao Chen, John Seon Keun Yi, Mark Kahoush, Erin S. Cho, Yong K. Cho

**Affiliations:** 1 Institute for Robotics and Intelligent Machines, Georgia Institute of Technology, 801 Atlantic Dr. N.W., Atlanta, GA 30332, USA; 2 School of Computer Science, Georgia Institute of Technology, 801 Atlantic Dr. N.W., Atlanta, GA 30332, USA; johnsk95@gatech.edu (J.S.K.Y.); mkahoush3@gatech.edu (M.K.); 3 Alpharetta High School, 3595 Webb Bridge Rd, Alpharetta, GA 30005, USA; sinahecho@gmail.com; 4 School of Civil and Environmental Engineering, Georgia Institute of Technology, 790 Atlantic Dr. N.W., Atlanta, GA 30332, USA; yong.cho@ce.gatech.edu

**Keywords:** laser scanning, point cloud, scene completion, building facades, occlusions

## Abstract

Collecting 3D point cloud data of buildings is important for many applications such as urban mapping, renovation, preservation, and energy simulation. However, laser-scanned point clouds are often difficult to analyze, visualize, and interpret due to incompletely scanned building facades caused by numerous sources of defects such as noise, occlusions, and moving objects. Several point cloud scene completion algorithms have been proposed in the literature, but they have been mostly applied to individual objects or small-scale indoor environments and not on large-scale scans of building facades. This paper introduces a method of performing point cloud scene completion of building facades using orthographic projection and generative adversarial inpainting methods. The point cloud is first converted into the 2D structured representation of depth and color images using an orthographic projection approach. Then, a data-driven 2D inpainting approach is used to predict the complete version of the scene, given the incomplete scene in the image domain. The 2D inpainting process is fully automated and uses a customized generative-adversarial network based on Pix2Pix that is trainable end-to-end. The inpainted 2D image is finally converted back into a 3D point cloud using depth remapping. The proposed method is compared against several baseline methods, including geometric methods such as Poisson reconstruction and hole-filling, as well as learning-based methods such as the point completion network (PCN) and TopNet. Performance evaluation is carried out based on the task of reconstructing real-world building facades from partial laser-scanned point clouds. Experimental results using the performance metrics of voxel precision, voxel recall, position error, and color error showed that the proposed method has the best performance overall.

## 1. Introduction

Point cloud data of buildings can be used for making 3D models for civil engineering applications [1]. These applications include urban mapping [2], building maintenance [3], energy analysis [4], and historic building preservation [5]. The building geometry represented in the point cloud format is a list of points in 3D space containing attributes such as 3D coordinates and RGB color. Point cloud data can easily be collected using terrestrial laser scanners (TLS), cameras, or Red-Green-Blue-Depth (RGBD) sensors. However, using the point cloud data is still problematic because of numerous sources of defects such as noise, occlusions, and moving objects [6]. The main source of these defects in both indoor and outdoor environments is occlusion. In indoor environments, occlusions are usually caused by furniture. Whereas in outdoor environments, scans of building facades are often occluded by trees and lamp posts. One way to fix the problem of having missing data is to capture scans from multiple locations and combine the scans together. However, this process is labor-intensive, time-consuming, and still may not fully collect the required data due to difficulty of access for data collection. Even with advanced scanning methods such as robotic scanning, the problem of occlusions and missing data remains a significant challenge.

Having an automatic point cloud scene completion method has several benefits, including improving data quality and reducing manual labor for data acquisition. Furthermore, point cloud completion has many application scenarios including reducing errors in registration [7], improving accuracy in object recognition [8], and improving quality for 3D modeling [9]. Several automated methods [8,10,11,12] exist in the literature but have their own limitations in terms of applicability to large, complex scenes and requirements for the input data. Many methods only work on point cloud data of small objects or data with only small, local-scale defects [10,11]. Other methods like ScanComplete [8] and SSCNet [12] predict the 3D geometry only and do not complete the color information. There exists a strong research need to explore methods that can reconstruct a complete 3D scene in terms of both geometry and color for large outdoor scenes such as building facades.

This research proposes a point cloud scene completion method for building facades using orthographic projection and generative adversarial inpainting methods. Since a scan of a building facade lies on a mostly two-dimensional surface, it is beneficial to convert the 3D unstructured data into a 2D structured representation that can encode neighborhood information. Thus, the raw 3D point cloud is first converted into a depth image and a color image using an orthographic projection. Next, a 2D inpainting method is applied to predict the location and color information of missing pixels in the converted image. The inpainting method consists of a deep neural network that is trained on a database with pairs of incomplete and complete images. Finally, the inpainted 2D image is converted back into a 3D point cloud by remapping the previously stored depth information. The output point cloud is evaluated by comparing it to the ground truth point cloud in a dataset of laser scans of building facades. The rest of the paper is organized in the following order: literature review, methodology, results, discussion, and conclusion.

## 2. Literature Review

### 2.1. Geometry-Based 3D Scene Completion

Existing geometry-based scene completion methods include mesh-based methods [10] and point-based methods [11,13,14,15]. Poisson surface reconstruction [10] is a well-known method that uses local interpolation methods to fit a mesh model to a defective point cloud, and a smoother version of the point cloud can be obtained by sampling from the mesh model. The surface reconstruction method works well for small holes but is unable to fix large holes since it only makes use of local geometry to compute the surface interpolation [8]. There are also other scene completion methods that are aimed at filling in holes in the object [13,15]. Hu et al. [13] enforce both intra-frame self similarity and inter-frame consistency to search for intra-frame self-similar cubes and inter-frame corresponding cubes. Adan et al. [15] does so by first visualizing the depth value using orthographic projection, then identifying holes in the depth-buffer and finally filling in the holes using meshes. These methods work well to restore holes with known sizes but may not work with irregular holes. Another option for planar surfaces such as the road or sidewalk is to use mathematical morphological operations such as dilation and connected components to fill in holes caused by occlusions [16,17]. A more advanced method uses a sequence of steps including wall detection, occlusion labeling, opening detection, and occlusion reconstruction to reconstruct a 3D model of an indoor environment from laser scans with missing data [15]. The method worked well for coarse and mainly planar building components but does not support environments with more complex geometries such as curved surfaces and non-rectangular openings [3].

Other geometry-based methods include As-is-BIM (Building Information Modeling) [18], which involve modeling the geometry of components, assigning an object category, and establishing relationships between components. Other methods such as Scan-to-BIM replace scanned geometry with Computer Aided Design (CAD) models retrieved from databases [19], while some methods find identical CAD models in a shape database for a given input shape and align it with the scans [20,21]. Yet, this relies on databases including identical shapes and matches for objects in 3D scans. Pauly et al. [22] use a prior in the form of a template model. The prior is then aligned with the acquired data and the holes are filled by the transfer of geometric data from the wrapped template. Apart from requiring a large shape database, it also needs consistent topology of the context models in the region in order to properly blend the prior and the data. The universal problem of using these latter methods is that they require additional inputs in the form of templates or models, and are unsuitable to be used for situations where only raw point cloud data is available.

### 2.2. Scene Completion with 2D Image Inpainting

Scene completion has also been widely explored in the 2D domain in the form of image inpainting. Image inpainting can be defined as the task of filling in missing or marked pixels in an image to create a contextually relevant result. It is widely used for completing holes or restoring damaged and occluded regions. Classical approaches to image inpainting focus on propagating information from neighboring pixels to the masked regions by using techniques like isophote feature propagation [23,24,25] and image mapping [26]. These approaches work well in inpainting small regions but suffer in large regions with varying textures. Other techniques used to resolve this drawback are exemplar based methods [27,28] and structure propagation [29]. Patch-based inpainting techniques focus on filling in missing regions by searching for replacement patches on the remaining parts of the image. Patches are usually sampled based on various distance metrics between patches such as scale invariant feature transform (SIFT) keys [30] and Euclidean distance. Various studies [31,32] have proposed speedups for the search process, including PatchMatch that proposes a randomized algorithm to quickly find approximate matches between image patches [33]. Hays and Efros [34] approached patching by finding and attaching a semantically valid region from an image dataset.

The use of convolutional neural networks (CNN) and deep learning for image inpainting brought seminal progress to image inpainting. Ren et al. [35] used a three-layer CNN to achieve promising inpainting results. Most deep generative models follow an encoder–decoder architecture. The encoder takes in an image with holes and produces a latent feature representation of the image. The decoder then decodes the latent features to produce an image. The context encoder [36] uses this architecture trained with an adversarial loss to produce contextually plausible results. However, it tends to lack detailed textures, and various later approaches [37,38,39,40] were developed to address this problem. Zeng et al. [41] used a pyramid context encoder on a U-Net [42] structure that learns region affinity from a high-level feature map to a low-level feature map in order to ensure both visual and semantic accuracy. Isola et al. [43] used a conditional generative adversarial network (cGAN) [44] to create realistic image-to-image translation results including inpainting.

One major limitation of these existing image inpainting methods is that manual annotation of a mask over the missing regions is required. Several later studies thus focus on eliminating the manual masking process. One study proposed a semiautomatic inpainting model that allows a user to specify some of the preselected candidates of potentially damaged regions [45]. Other approaches [43,46,47,48] automatically select the damaged or lost regions guided by structural information or a model of common forms of damage or alterations. However, these methods are mainly applied for 2D images and have not been evaluated in the context of 3D point clouds.

### 2.3. Data-Driven 3D Scene Completion

Recent advances in deep learning have created opportunities for the approaches to automated shape generation and scene completion that are more data-driven as opposed to purely geometry-based. Earlier works focus on single object shape completion [49,50,51,52]. However, to apply these methods, additional segmentation is required to extract individual objects from a larger scene. Recently, methods using deep learning have been developed [8,9,12,49,50,51,52,53], and some extend beyond single objects to perform semantic scene segmentation [8,9,12,53]. One approach focused on point cloud upsampling [50], creating denser and more uniform sets of points from a sparse input. Another approach uses a 3D encoder–predictor convoluted neural network that combines a 3D deep learning architecture with a 3D shape synthesis technique [51]. Whereas, other approaches complete scenes by inferring points from depth maps [12,53]. ScanComplete [8]—a large-scale scene completion method from 3D scans—uses fully convoluted neural networks that can be trained on smaller sets and applied to larger scenes, which allows for efficient processing. To develop geometrically dense outputs, the model predicts a multiresolution hierarchy of outputs, where each hierarchy level makes use of information from the previous hierarchy level. However, it requires truncated signed distance field (TSDF) information as an input and operates on dense volumetric grids, which significantly limits its output resolution [9]. In addition, despite the encouraging results of the above methods, their applications are mostly limited to interior areas or objects rather than exterior ones.

Other point cloud completion methods that use point-based neural networks for scene completion include point completion network (PCN), TopNet, and FoldingNet [7,54,55]. These methods commonly use an encoder such as PointNet [56] that combines point-wise multilayer perceptrons with a symmetric aggregation function to compute both point-wise and global features. In PCN [7] an encoder–decoder network that maps partial shapes to complete shapes is used. PCN does not retain the input points in its output; instead, it learns a projection from the space of partial observations to the space of complete shapes and creates an entirely new point cloud. Similarly, TopNet [54] uses a general decoder that can generate structured point clouds by implicitly modeling the point cloud structure in a rooted tree architecture. AtlasNet [57] further extends the decoding process by representing the 3D shape as a collection of parametric surface elements and infers a surface representation of the shape. The main limitations of these methods are that they do not predict color information of the points, they do not preserve the scale and offset as an entirely new point cloud is generated, and they only predict a constant number of points and, therefore, is less scalable to large scenes [54]. It is harder for these methods to predict detailed geometry because the output is unstructured, and, as such, they are designed for single objects and not for complex scenes with multiple building elements with obstructions.

In view of these limitations, this research proposes a novel projection and inpainting method for the case of point cloud scene completion of building facades. In contrast to many works in the literature, which only consider 2D images or small 3D objects, this research will consider large, complex 3D scenes. The proposed method can work directly with point cloud data, is able to reconstruct color information, and can also work for curved surfaces. In addition, the generative adversarial inpainting network is data-driven and trainable end-to-end, which makes the method highly automated and can be implemented without significant manual annotation or tuning.

## 3. Methodology

**Figure 1:**
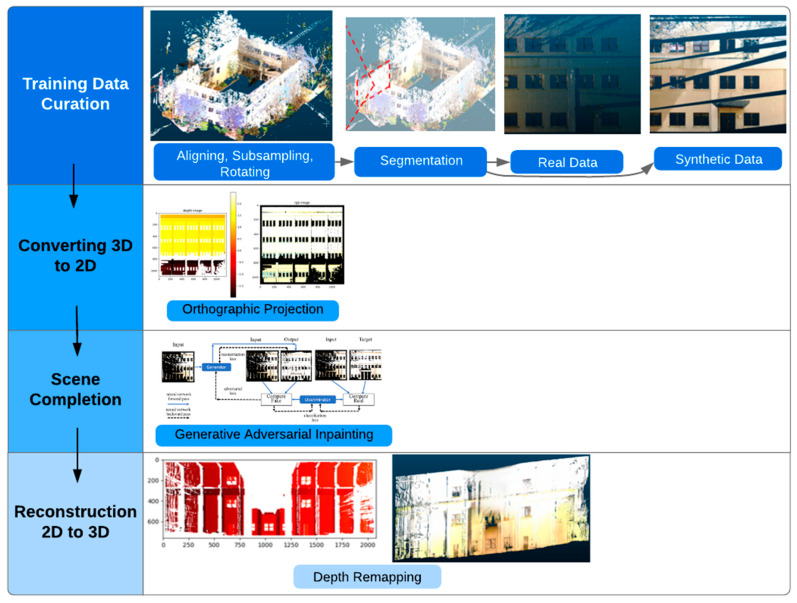
Workflow diagram for point cloud scene completion using orthographic projection and generative adversarial inpainting.

The proposed method for scene completion consists of first preprocessing the 3D point cloud, converting the 3D point cloud into 2D representations, then using data-driven methods to predict the geometry and color of missing regions in the scene, and finally converting the 2D representation back into a 3D point cloud. Figure 1 shows the overall workflow for the proposed method. Each step is explained in detail in the following subsections.

### 3.1. Point Cloud Data Preprocessing

**Figure 2:**
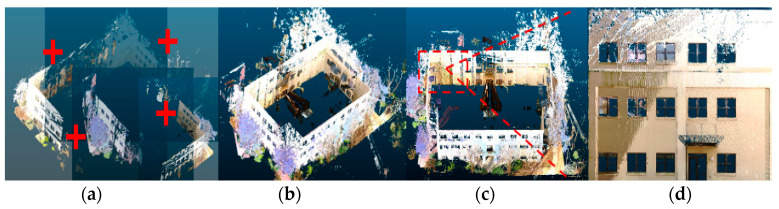
Example of preprocessing steps applied to a building point cloud: (**a**) registration, (**b**) downsampling, (**c**) rotation, and (**d**) final extracted building facade.

This section describes how the raw point cloud data was preprocessed into a standardized format that could be used for scene completion. For each building facade, the laser scans from different scan positions were manually registered so that they lay in the same coordinate system. The scans were also rotated so that the building point cloud was aligned with the X–Y axes. In addition, the scans were downsampled using a voxel grid filter. Voxel grid downsampling had the added effect of equalizing resolution throughout the entire point cloud since the resulting points were evenly spaced according to the voxel grid. Figure 2a–c shows the preprocessing steps including registration, downsampling, and rotation carried out on the raw point cloud data using CloudCompare [58]. Finally, a building facade was extracted from the scene by extracting a single side of the building and removing any surrounding clutter. Figure 2d shows the final extracted point cloud of a building facade. Note that these preprocessing steps were carried out manually for convenience in this study but can be easily automated using point cloud processing libraries.

The input and ground truth point clouds for the purposes of evaluating scene completion were generated according to the following rule: (i) the input point cloud is obtained from an individual partial scan of a building facade, which is incomplete, and (ii) the ground truth point cloud is obtained from the combination of all scans of the same building facade, which is usually more complete. Note that the combinations of all scans may still be an imperfect representation of the scene but they were considered as the ground truth because it had the most available information.

**Figure 3:**
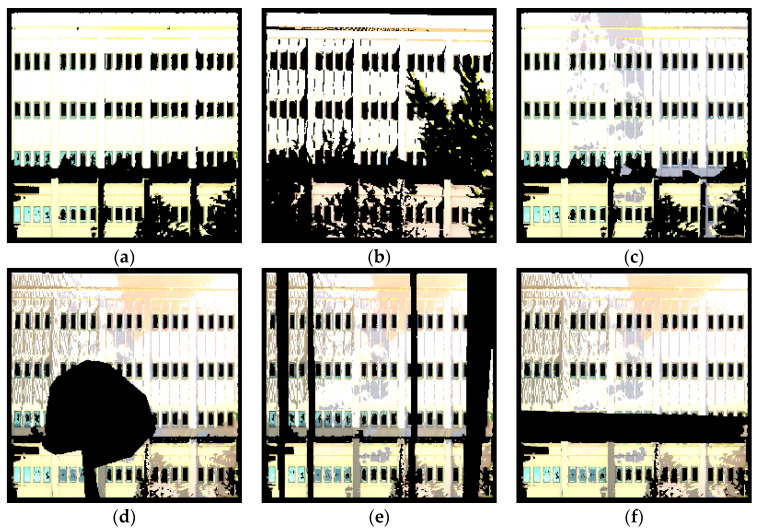
Examples of augmented training data with (**a**–**c**) scan recombination with real occlusions and (**d**–**f**) data with synthetic occlusions.

The point cloud data was also further processed to generate training data for the learning-based scene completion method that will be described in Section 3.2.2. In order to generate sufficient data to train a network that can be robust to different types of defects, the technique of data augmentation was used in this study. First, scan recombination was used to create multiple input scans from different combinations of a set of input scans (e.g., by combining scans #1 and #2, then combining scans #2 and #3, then combining scans #1, #2, and #3, etc.). Next, synthetic occlusions were created by randomly removing sections from the point cloud data. This was accomplished through the use of a segmenting tool and manually extracting shapes of common sources of occlusions such as trees and lamp posts. Figure 3 shows different variations of training data that were generated for the same building facade. Using these techniques, around 40–50 additional training data could be generated for each scene.

### 3.2. Scene Completion with Orthographic Projection and Generative Adversarial Inpainting

#### 3.2.1. Point Cloud to Image Conversion Using an Orthographic Projection

**Figure 4:**
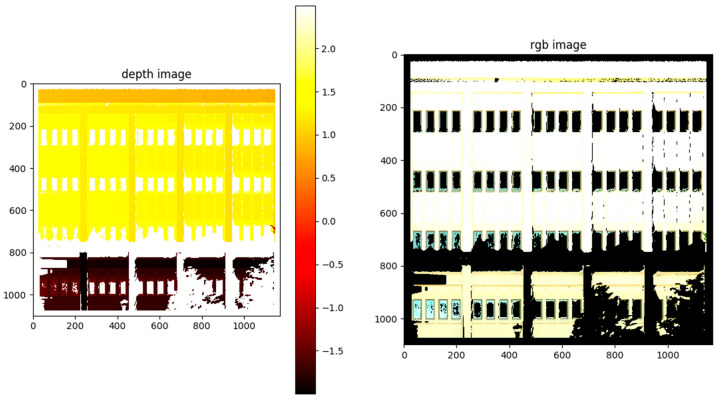
Visualization of the orthographic projection of a point cloud to create a depth image (**left**) and an RGB color image (**right**). The depth image is shown as a heat map with units of depth in meters. Units on horizontal and vertical image scales are pixels.

This study used a point cloud orthographic projection to convert the 3D point cloud scene to 2D image representations. An adaptation of a third party implementation [59] was used for this process. Given the point cloud coordinates, the center and half XYZ-extent (distance from center to the edges of the point cloud) were first calculated. Using this information, a bounding box was then created around the points. The X, Z coordinates of the points inside the box were normalized between 0 and 1. Whereas, the Y coordinates were preserved to retain depth information. Then, the size of the bounding box was scaled by a predetermined density value to create a scaled XZ-plane. A larger density value will result in a larger and more detailed 2D projection but will also result in increased computation time in later processing steps. In this study, the density values were set to around 50–80 pixels per m^2^ by trial and error to balance image quality while saving computation time. The RGB values for each point in the point cloud were projected to the corresponding space in the scaled plane. Equations (1) and (2) below show the calculation of image coordinates based on the projection from point cloud coordinates. Whereas, the depth information (i.e., Y coordinates) of the points were projected in a similar manner and stored separately (Figure 4). Lastly, a median filter was applied to the RGB image and the depth image to reduce noise. The RGB image was used in image inpainting, whereas the depth image was used for depth recovery in a later step.

(1) $$x_{image}=\frac{density\cdot x_{point\;cloud}\;}{x_{bounding\;box}}$$

(2) $$z_{image}=\frac{density\cdot z_{point\;cloud}\;}{z_{bounding\;box}}$$

#### 3.2.2. Fully Automated 2D Inpainting with Generative Adversarial Networks (GANs)

In this research, we proposed a fully automated method for 2D image inpainting using a customized generative adversarial network (GAN) based on Pix2Pix [43]. Pix2Pix takes the form of a conditional adversarial network consisting of a generator and a discriminator that can learn the mapping from an input image to an output image. Unlike other networks that are tailored to work on a specific case of image translation, Pix2Pix offers a generic solution that can be applied to different image-to-image translation tasks. This is advantageous because the networks can be trained end-to-end, and the discriminator can learn a loss function to train the generator, eliminating the need to manually fit loss functions for different tasks.

The dataset used to train the network consists of pairs of an input image, which represents the incomplete scene, with a target image, which represents the ground truth (more complete scene). By analyzing patterns in the training data, the generator can learn how to fill in incomplete regions in the input image. The discriminator then used the generator output and tried to differentiate between the artificial image that was generated and the real target image. This conditioned the generator to also output more visually realistic images.

**Figure 5:**
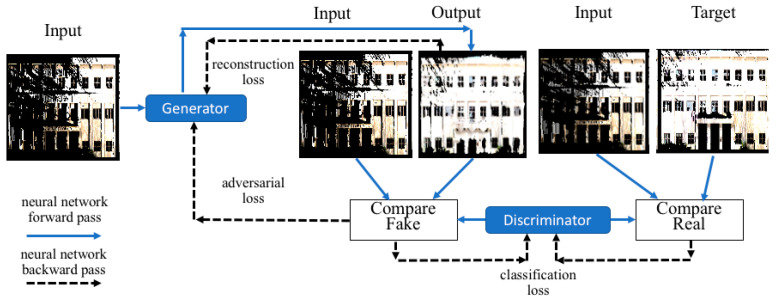
A framework of training the generator and the discriminator networks for 2D inpainting.

The framework for training the generator and discriminator networks is shown in Figure 5. The generator was trained based on the pixel-wise reconstruction loss between the input and target images. Whereas, the discriminator was trained by examining the target image associated with the input/target pair as well as the output image from the generator and classifying whether they were real or synthetic. Finally, the generator’s weights were then additionally adjusted according to the adversarial loss function such that the discriminator was unable to differentiate the output and target images as to whether they were real or synthetically generated.

**Figure 6:**
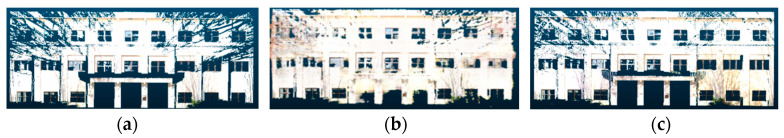
Example of fully automated 2D inpainting with a generative adversarial network: (**a**) input scan, (**b**) building facade after inpainting, and (**c**) target ground truth.

To train and evaluate our model on our dataset, we used the method of leave-one-out cross validation. This is a form of K-fold cross validation where K was equal to N (the number of samples in the dataset). This means that every time the network was trained, the test data was obtained from one scene, whereas the training data was obtained from all the remaining N-1 scenes. In the case of the dataset used in this paper, we used one scene for testing and the remaining ten scenes for training. This allows the training process to maximize the amount of training data available while maintaining the separation between training data and test data. Figure 6 shows an example of the inpainting results using the proposed method.

#### 3.2.3. Image to Point Cloud Conversion Using Depth Remapping

The image inpainting output, whether using semiautomated or fully automated inpainting, was in a 2D image form and needs to be converted back to a 3D point cloud to obtain the desired result. For this, we used the depth information of the original point cloud that was previously stored when performing the orthographic projection in Section 3.2.1. The X and Z coordinates could be easily recovered since they exactly corresponded to the scaled pixel positions of the 2D image. Density and bounding box information was retrieved from the saved parameter file and used to scale the coordinates back to the dimensions of the original point cloud. Depth information (i.e., the Y coordinate) could be directly recovered for points that already existed before inpainting.

**Figure 7:**
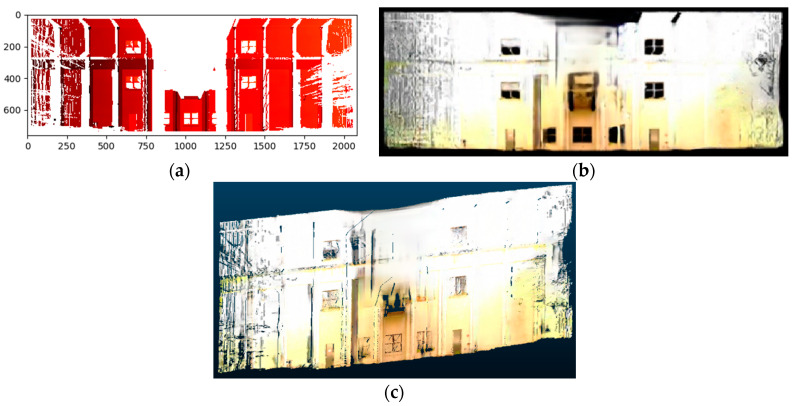
Visualization of the image to point cloud conversion process: (**a**) depth information of the original image, (**b**) a filled image using depth information, and (**c**) a resulting 3D point cloud.

To predict the depth of the newly filled pixels, we used the nearest neighbors method to find the nearest pixel in the original depth image and set the missing depth to that of the nearest pixel. In the general case, it is true that depth remapping can have errors when the depth of the obstructed area is different from the depth of the neighboring areas. However, in our case of building facades, the scene did not have arbitrary depth but was well structured with many repeating patterns. For example, when considering a flat planar section of a wall, it is usually the case that a small section of the wall and not the entire wall is occluded; thus, we could reconstruct the depth of the occluded part of the wall using the depth of the non-occluded part of the wall. For depth remapping, we used a k-d tree (K-dimensional tree) data structure, implemented in the Fast Library for Approximate Nearest Neighbors (FLANN) library [60], to speed up the search process for nearest neighbors. During this process, noisy pixels in the filled image were also filtered out by applying a brightness threshold. Finally, the resulting set of XYZRGB points was saved as a point cloud in the PLY format. Figure 7 shows a visualization of the intermediate results and the final point cloud created using depth remapping.

## 4. Results

### 4.1. Composition of the Dataset

**Figure 8:**
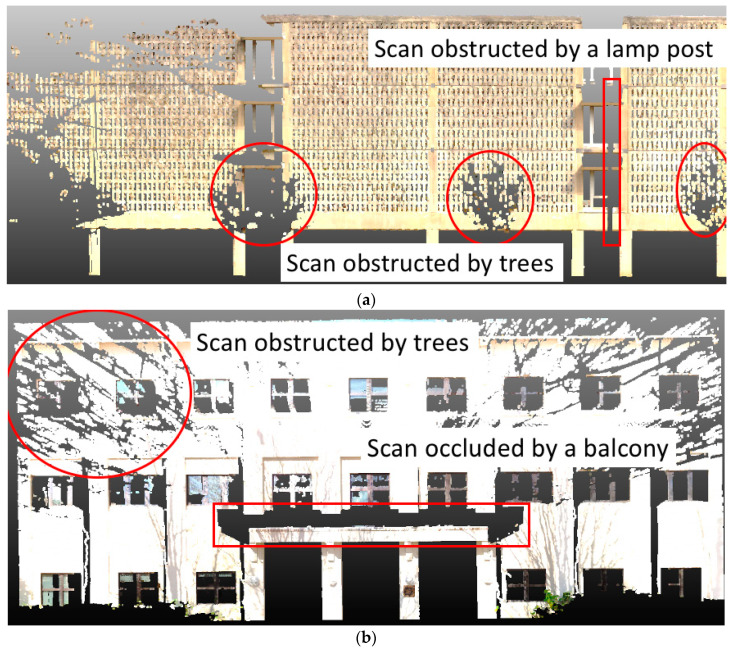
Example defective point clouds contained in the proposed dataset. (**a**) example of a scan obstructed by trees and a lamp post, (**b**) example of a scan obstructed by trees and a balcony

The point cloud data used in this research was taken from laser scans of different buildings on the Georgia Tech campus. An average of 3–5 scans was taken for each building facade. There were 11 building facades in total that were used for testing. As shown in Figure 8, the individual scans contained numerous defects caused by obstructions from trees, lamp posts, and building structures. Thus, it was an effective dataset to evaluate the ability of different scene completion methods to fix defects in point cloud data. In contrast to existing datasets in the literature for scene completion (e.g., SunCG [12] and ScanNet [61]), which are mostly indoor scans with RGBD sensors, our dataset used a terrestrial laser scanner (TLS) and focused on large, outdoor scenes.

### 4.2. Scene Completion Baseline Algorithms

This section will present four baseline algorithms for point cloud scene completion that will be used for comparison against the proposed method, namely (i) Poisson reconstruction [10], (ii) hole filling [62], (iii) plane fitting [63], and (iv) partial convolutions [64]. Poisson reconstruction, hole filling, and plane fitting are geometry-based scene completion methods whereas partial convolutions are a data-driven image-based scene completion method. An additional baseline algorithm combining hole filling and Poisson reconstruction was also considered for evaluation purposes (discussed in the next section). Note that these methods had some limitations when applied for the scene completion task but could still serve as useful comparisons for the proposed method.

#### 4.2.1. Baseline I: Poisson Reconstruction (Meshing)

Poisson reconstruction works by creating a filled mesh over existing point cloud data. The input data was a set of points, each containing its coordinates in a 3D volume, its RGB values, and its inward-facing normals, whereas the target output was a triangulated approximation to the surface. Initially, based on the fact that the normal field of the object can be interpreted as the gradient of the solid’s indicator function, the surface integral can be constructed. Next, the indicator function was reconstructed from this gradient field by formulating it as a Poisson problem. This led to a reconstructed watertight, triangulated approximation to the surface by approximating the indicator function of the model and extracting the isosurface. Finally, points were sampled from the resulting mesh to obtain the surface approximation represented in the point cloud. This method exported the normals and color information of the original mesh, through interpolating the information in each triangulated surface, to obtain the final point cloud. The implementation used in this study was from the CloudCompare software [58], which internally uses the algorithm from [10].

#### 4.2.2. Baseline II: Hole Filling

The hole filling process works as follows: given a set of input points containing coordinates in 3D space, and their RGB values, the output is the original set of points with additional synthetic points that cover the holes. The points were first normalized by shifting the points to the origin in one axis. Next, the neighboring points were connected to form triangles in the 3D scene. Larger triangles were identified by computing the circumradius and area. These larger triangles were then converted to polygons, which represent the holes in the input scene. A size threshold was used to differentiate between holes that were caused by occlusions and holes that were naturally present in the scene. To achieve this, a minimum size cutoff was used to prevent the hole filling step from completely filling in windows and other openings that were naturally present in the scene. Note that this cutoff procedure led to the side effect that some holes caused by occlusions were filled whereas others were not. Once the locations of the holes were identified, we could generate the X,Y,Z coordinates of the synthetic points that were to be added to the scene. These points were evenly distributed within the extent of each hole. Once the synthetic points were generated, the points outside of the bounding shape were clipped. Whereas, the points inside of the bounding shape were preserved and assigned an RGB color value based on the average RGB value of the input data. The implementation used in this study was from an open-source algorithm for hole detection and filling [62].

#### 4.2.3. Baseline III: Plane Detection and Fitting

Since most buildings consist of flat planes, plane fitting is a reasonable method to use for scene completion. Several works in the literature also take advantage of local planarity to reconstruct 3D point clouds [2,13,15]. The plane detection step works by randomly selecting sets of three points and using singular value decomposition to find the plane parameters that fit the selected points. The random sample consensus (RANSAC) method [63] was used in this step to find the optimal plane and account for outliers. Once a plane was found, points were assigned by uniformly filling in gaps in the plane. The color of the newly filled points was set to the mean of the points in the plane. The same process was repeated for the remaining regions in the scene until all planes were accounted for. For this implementation, we repeated the plane detection and filling process until either 100 planes were found or the number of remaining points was less than 500. For RANSAC, we set the number of iterations to 100 and the inlier threshold to 0.05 m. These parameters were obtained by trial and error on the test data.

#### 4.2.4. Baseline IV: Masked 2D Inpainting with Partial Convolutions

Given the converted 2D building images, we could apply a semiautomated method for image inpainting. The input image was annotated with a user-defined mask over the incomplete regions and processed using a neural network with partial convolutions [64]. Unlike other inpainting methods that used convolution on both masked and unmasked regions, partial convolution applies only to valid pixels. The model utilized a U-Net like architecture similar to the one used in [43] with all convolutional layers replaced with partial convolutional layers. In this study, the masks were manually annotated and processed using the publicly available image inpainting implementation provided in [65], which utilizes a pretrained network.

### 4.3. Scene Completion Performance Evaluation

The performance of each point cloud scene completion algorithm was evaluated using laser-scanned point clouds of building facades acquired at the Georgia Tech campus (described in Section 4.1). The evaluation metrics were (i) voxel precision, (ii) voxel recall, (iii) F1-score, (iv) position root-mean-squared-error (RMSE), and (v) color root-mean-squared-error (RMSE). Voxel precision and recall were calculated by projecting the point cloud onto a uniform voxel grid at 0.05 m resolution and comparing the output result with the ground truth. The F1-score was then computed by taking the harmonic mean of the precision and recall scores. The position RMSE was calculated by taking the point-wise difference in XYZ coordinates between the output and ground truth point clouds over all matched voxels. Since the output from scene completion was in the same coordinate system as the input and ground truth, the voxel grids could be matched directly to find point correspondences. To be precise, the formula for calculating position RMSE is:

(3) $$\mathrm{RMSE}=\sqrt{\sum_{i=1}^{n}\frac{{\left(\hat{y_{i}}-y_{i}\right)}^{2}}{n}}$$

where ${\hat{y}}_{1},{\hat{y}}_{2},\;\ldots ,{\hat{y}}_{n}$ are the predicted XYZ coordinates, $y_{1},y_{2},\ldots ,y_{n}$ are the ground truth XYZ coordinates, and n is the number of common voxels. Note that the position RMSE does not take into account predicted voxels with no ground truth voxel, but the frequency of these false positive occurrences can be measured using the voxel precision score. Whereas, the color RMSE is calculated in a similar manner to the position RMSE, but using the RGB channels normalized from 0 to 1.

To evaluate the proposed method, we trained our inpainting network with the building facade dataset using the cross-validation technique discussed in Section 3.2.2. The network was trained for a total of 100 epochs with a batch size of 10 images and a learning rate of 0.002. We also implemented several improvements to the original Pix2Pix network. First, in order to increase the diversity of data available for training the model, we used the technique of data augmentation. As discussed in Section 3.1, this was performed by registering different combinations of scans of the same scene as well as creating synthetic occlusions in the input scans. In total, our network was trained on a dataset of 467 images, of which 180 were obtained from synthetic occlusions, and 287 were obtained from registered scans with real occlusions. Next, feature augmentation was applied to sparse regions in the image by appending the RGB color information from the nearest neighbor pixel. This was to increase the number of input features available to the generator when creating reconstructed images. In addition, a color smoothing operation was applied to the output image by updating the RGB color of filled-in pixels with an average of 10 nearest neighbor pixels from the input image.

Table 1 shows a comparison of the scene completion accuracy metrics, averaged across the 11 test sets, where each test set was a single building facade. The proposed method using orthographic projection and generative adversarial inpainting was compared against four other baseline methods, which were (i) Poisson reconstruction, (ii) hole-filling, (iii) hybrid method combining Poisson reconstruction and hole-filling, and (iv) plane-fitting. Results show that the proposed achieved the highest voxel recall, F1-score, and position RMSE. The proposed method also achieved good results for voxel precision and color RMSE.

**Figure 9:**
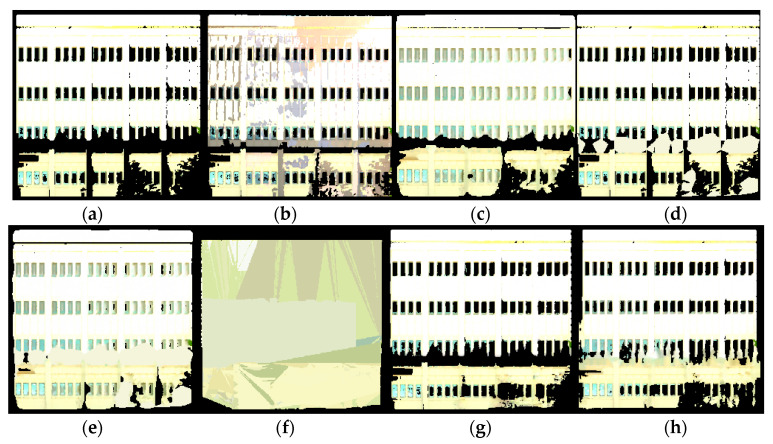
Point cloud scene completion results for the Mason building, visualized in 2D: (**a**) input point cloud, (**b**) target ground truth, (**c**) Poisson reconstruction, (**d**) hole-filling, (**e**) hybrid, (**f**) plane-fitting, (**g**) partial convolution, and (**h**) proposed.

**Figure 10:**
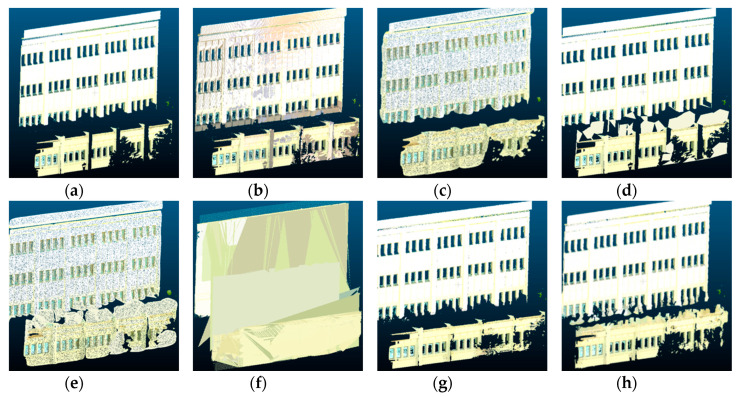
Point cloud scene completion results for the Mason building, visualized in 3D: (**a**) input point cloud, (**b**) target ground truth, (**c**) Poisson reconstruction, (**d**) hole-filling, (**e**) hybrid, (**f**) plane-fitting, (**g**) partial convolution, and (**h**) proposed.

Figure 9 and Figure 10 show a comparison of the scene completion results for the Mason building at Georgia Tech, visualized both in 2D and 3D. In this scene, the gap between the upper and lower sections of the building was especially challenging. The Poisson reconstruction method did not fill in this gap, whereas the hole-filling method and plane-fitting methods filled in the gap incorrectly.

**Figure 11:**
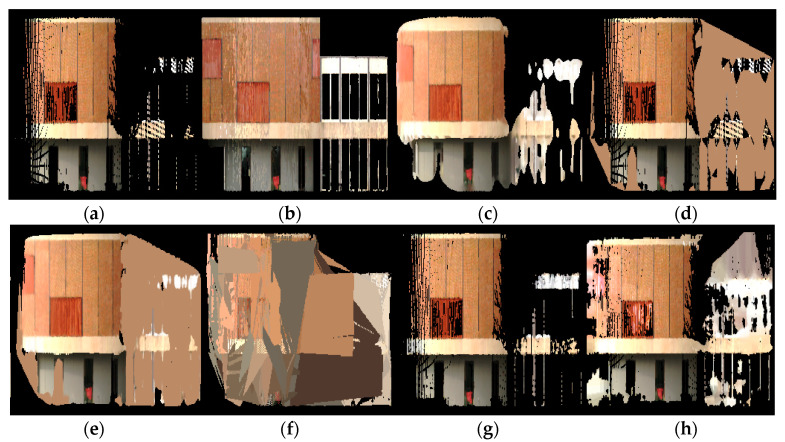
Point cloud scene completion results for the Van Leer building, visualized in 2D: (**a**) input point cloud, (**b**) target ground truth, (**c**) Poisson reconstruction, (**d**) hole-filling, (**e**) hybrid, (**f**) plane-fitting, (**g**) partial convolution, and (**h**) proposed.

**Figure 12:**
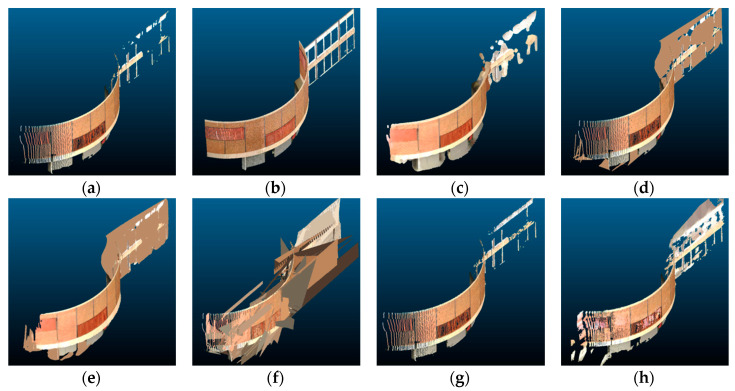
Point cloud scene completion results for the Van Leer building, visualized in 3D: (**a**) input point cloud, (**b**) target ground truth, (**c**) Poisson reconstruction, (**d**) hole-filling, (**e**) hybrid, (**f**) plane-fitting, (**g**) partial convolution, and (**h**) proposed.

Figure 11 and Figure 12 show a comparison of the scene completion results for the Van Leer building at Georgia Tech, which was especially challenging because it contains a section of the curved wall as well as a section of the planar wall. The curved wall demonstrated the weakness of the plane-fitting method, which only works for flat planes. The planar section was also difficult to reconstruct because the input was severely occluded in that region. The Poisson reconstruction and partial convolution methods did not fully reconstruct the region, whereas the proposed method performed better at reconstructing the region with relatively minor errors.

**Figure 13:**
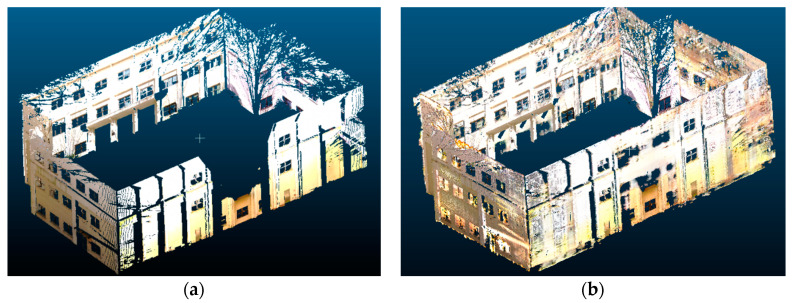
Example of 3D scene completion of a full building from partial scans: (**a**) input point cloud and (**b**) point cloud result after orthographic projection and generative adversarial inpainting.

Figure 13 shows an example of 3D reconstruction from partial scans for the sustainable energy building (SEB) at Georgia Tech. The input point cloud was taken from four separate laser scans of the building, one from each side of the buildings. From Figure 13a, the point cloud had several missing sections in each wall due to occlusions from trees, poles, and building structures. The proposed orthographic projection and generative adversarial inpainting method was applied to obtain a more complete version of the point cloud scene. The building was first divided into four separate facades, and each of them was converted into 2D image format using orthographic projection. Note that each facade was associated with a registration offset, but the offset was removed during the orthographic projection step. Next, each 2D image was processed using our trained neural network to predict how to fill in the missing regions. Finally, the 2D images were converted back into 3D point clouds using the depth remapping procedure. The separate facades were merged back into the original building coordinate system by applying the previously stored registration offsets. As shown in Figure 13b, the resulting point cloud had significantly fewer missing sections compared to the original point cloud and was a more visually coherent 3D representation of the building. More importantly, the proposed method was able to recover window patterns in the front facade of the building, showing that these geometric patterns could be learned in a data-driven manner.

**Figure 14:**
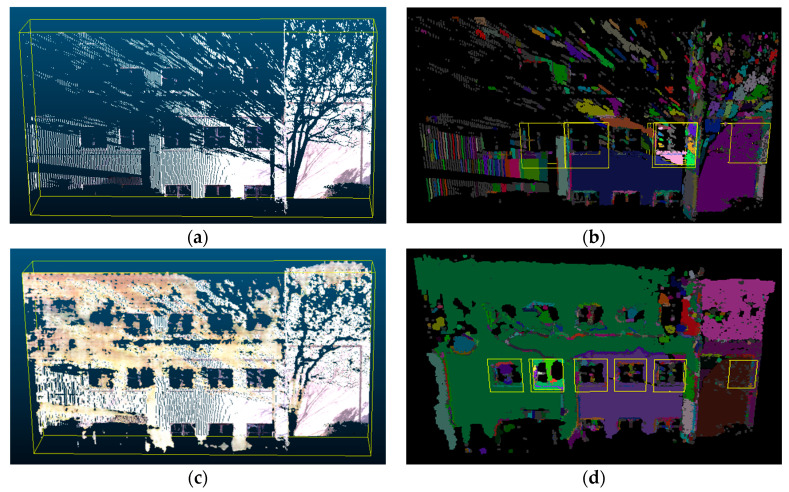
Example application scenario of using building facade completion to improve building element recognition results: (**a**) input point cloud, (**b**) window retrieval using the input point cloud, (**c**) output point cloud after generative adversarial inpainting, and (**d**) window retrieval using the output point cloud.

Figure 14 shows an example application scenario where building facade completion could improve the performance of scene semantic parsing algorithms such as building element recognition. The exemplar-based retrieval algorithm from [5] was used to perform window retrieval on two different point clouds: (i) the original incomplete point cloud and (ii) the completed point cloud after applying the proposed generative adversarial inpainting method. As shown in Figure 14, the window retrieval algorithm could only detect three windows correctly (with one incorrect detection) using the original point cloud due to the high amount of occluded regions and the low number of points in the occluded regions. After applying automated scene completion, the performance of the window retrieval algorithm improved and it could detect five windows correctly (with one incorrect detection).

## 5. Discussions

**Figure 15:**
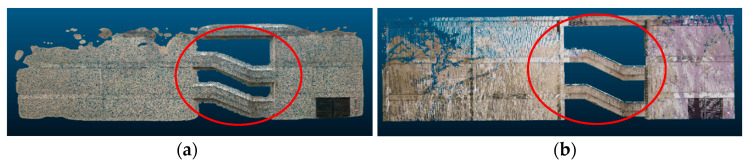
Example of a Poisson reconstruction limitation. (**a**) Side of a building after reconstruction and (**b**) target ground truth.

This discussion section was aimed at qualitatively comparing the performance of different types of scene completion methods and analyzing the effect of different hyperparameters used in this study. The strengths, weaknesses, and limitations of each method was first discussed in the following subsections:

Poisson reconstruction: the advantage of using Poisson reconstruction is that it smoothly reconstructs the surface of the buildings even in the presence of noise, and it usually completely fills in smaller occlusions. A limitation of Poisson reconstruction is that it may not preserve original geometric information in the scene. As seen in Figure 15, when Poisson reconstruction was applied (left), the openings between each floor level (red circle) became smaller, even though they should not change size as they were not caused by occlusions but rather were part of the building. Additionally, a common challenge in surface reconstruction is the recovery of sharp features, and Poisson reconstruction tends to incorrectly smoothen the sharp edges of the buildings.

**Figure 16:**
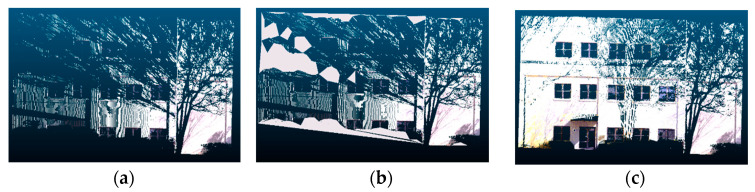
Example of a hole-filling limitation. (**a**) Input, (**b**) building facade after hole-filling, and (**c**) target ground truth.

Hole filling: this method works well for simple walls with a few larger occluded regions but does not perform well in the environments with more complex geometries with irregular-sized openings. The method relies on a size threshold parameter to differentiate between openings caused by occlusions and openings that are naturally present in the scene. Thus, when it comes to smaller holes, the hole filling algorithm may not correctly detect them as valid openings to be filled. As seen in Figure 16, when the hole filling algorithm was applied (middle), mostly the larger occlusions were filled, whereas the smaller ones were ignored.

Hybrid: this method combines the previous two algorithms by applying the hole-filling procedure and then applying Poisson reconstruction. The intuition is that the hole-filling step could fill in larger holes in the scene, whereas Poisson reconstruction could fill in smaller holes in the scene. However, the downside of this approach is that the combined process is too aggressive in filling in holes in the scene. As a result, even natural openings and empty spaces that are originally present in the scene are covered up in the output point cloud. This can be observed in Figure 10 and Figure 12, where the scene completion results for the hybrid method had many false positive points in regions that were supposed to be empty space in the ground truth point cloud.

Plane fitting: this method has the advantage that it relies on a simple geometric model and can be quickly implemented and computed. However, this leads to the limitation that it relies on the closeness of fit and generally sacrifices object details for the speed of reconstruction [66]. As can be observed in the result figures in the previous section, building facades were usually not completely flat but could have different widths at each floor as well as multiple protruding sections. Figure 12 shows a clear example where the plane fitting method did not work, which was a building with a curved wall.

Partial convolutions: in contrast with the previous methods that are more geometry-based, this inpainting method performs scene completion by learning a mapping from incomplete inputs to complete outputs in a data-driven manner. The strength of data-driven methods is that they are more robust to variations of the input data and rely less on preset threshold parameters but can be trained to adapt to different types of data defects. Results in Table 2 show that this method with partial convolutions worked well overall. The advantage of using this method with partial convolutions is that the convolution operation is conditioned only on valid pixels, so it performs better for irregular holes. However, the limitation of this method is that it is only semiautomated since it requires manually specifying an input mask over the incomplete regions.

**Figure 17:**
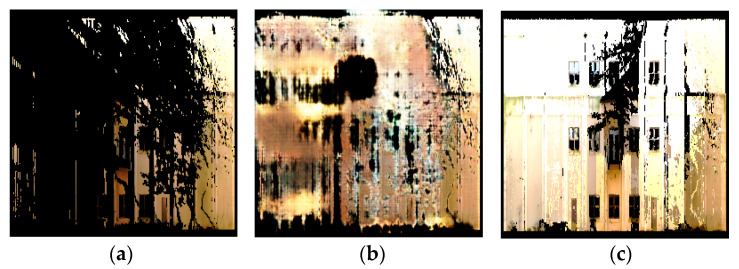
Example of a Pix2Pix limitation: (**a**) input, (**b**) building facade after applying Pix2Pix, and (**c**) target ground truth.

Proposed method with generative adversarial inpainting: one advantage of using Pix2Pix as the base network for 2D inpainting is that it uses a U-Net architecture instead of an encoder–decoder architecture. This allows for lower-level information to propagate to higher layers of the network, ultimately improving the visual cohesiveness of the results. However, the quality of the output for sparse inputs is still problematic in certain regions. This is because the generator does not enforce global consistency, and therefore may not produce output images with consistent color, pattern, and texture. As seen in Figure 17, after applying Pix2Pix (b) on a sparse input, the resulting image had uneven quality, texture, and color when compared to the target ground truth (c).

To partially address this limitation, this study made several modifications in our implementation compared to the original Pix2Pix network to improve its performance on the scene completion task. First, the original incomplete image was very sparse due to large regions with missing information. To overcome this, feature augmentation was applied to append three augmented feature channels to the input image in addition to the original RGB color channels. The augmented feature channels were obtained by taking the RGB color from the nearest neighbor pixel that had a non-null value. Next, the output pixels were generated as independent predictions from the Pix2Pix network even though there should be local correlations between neighboring pixels to generate a smooth image. To address this, a color smoothing operation was applied to the output image by updating the RGB color of filled-in pixels with an average of 10 nearest neighbor pixels from the input image. The improvement in the scene completion performance metrics after implementing each modification is summarized in Table 2.

For future work using generative adversarial networks for inpainting, further steps can be taken to improve the scene completion results. The major component to be improved is the quantity and quality of the training data. This research uses ten building scenes to train the network and one scene to test. Adding more building scenes in the training data will improve the network’s ability to generalize and fill in differently shaped defects in the building facades. Improving the quality of our ground truth data will also help train the Pix2Pix network better since some of the current ground truth point clouds still contain occluded regions. This can be resolved by using a more cutting-edge 3D scanner, collecting more scans to eliminate holes, or synthetically filling in the missing regions.

Aspect ratio ablation study: in resizing the images to fit the input size of the inpainting methods discussed in Section 3.2.2, this study resized the images without preserving the aspect ratio of the original image. This caused the resized input images to appear horizontally compressed, especially for rectangular building facades with long widths. Figure 11 shows some examples of compressed images due to the altered aspect ratio. Due to the concerns that ignoring the aspect ratio will result in lost features when performing inpainting and appear blurry when resized back to the original image dimensions, we analyzed the difference in performance when preserving the aspect ratio compared to when ignoring the aspect ratio. Table 3 demonstrates the results of an accuracy comparison on part of the test dataset. Four buildings that have rectangular facades were chosen for this analysis. The averaged F1-score was slightly better when having the aspect ratio preserved, whereas the color RMSE was better when ignoring the aspect ratio. However, these changes were too marginal to affect the overall performance. Thus, in the final evaluation, the authors used the variant that ignored the aspect ratio due to the simpler implementation.

Depth remapping ablation study: in the depth remapping step, depth values were assigned to newly filled points. This research used nearest neighbors to assign the depth of the closest neighboring point. Table 4 below shows the accuracy results of filled point clouds when more than one nearest neighbor was used for depth assignment. In the case below, one, two, or ten nearest neighbors were chosen and the average of their depth values was assigned to the new point. The results demonstrated that the 1-Nearest Neighbor setting had the best performance overall in terms of the voxel precision, voxel recall, and color RMSE.

Voxel size ablation study: when computing the accuracy metrics such as voxel precision and voxel recall, an important parameter that is involved is the voxel size. Results in Table 5 show that changes in voxel size changed the absolute value of each performance measurement, but the relative order of rankings of the methods remained mostly the same. With a lower voxel size, the accuracy metrics generally decreased, while with a higher voxel size, the accuracy metrics generally increased. For each voxel size, the proposed method still achieved the highest F1-score.

In general, a smaller voxel size allows the accuracy metrics to capture whether the scene completion methods are able to reconstruct fine-level detail in the point clouds. A smaller voxel size imposes a stricter criterion for matching the predicted point cloud to the ground truth point cloud when computing the voxel prediction and recall. For example, a voxel size of 0.01 m means that a predicted point has to be within 0.01 m of a ground truth point in order to be considered a true positive whereas a voxel size of 0.2 m means that a predicted point can be within 0.2 m of a ground truth point and still be considered a true positive. In addition, using small voxel sizes also cause the metrics to be more sensitive to noise in the input data that is caused by the sensing device and not due to errors in the scene completion algorithm.

**Figure 18:**
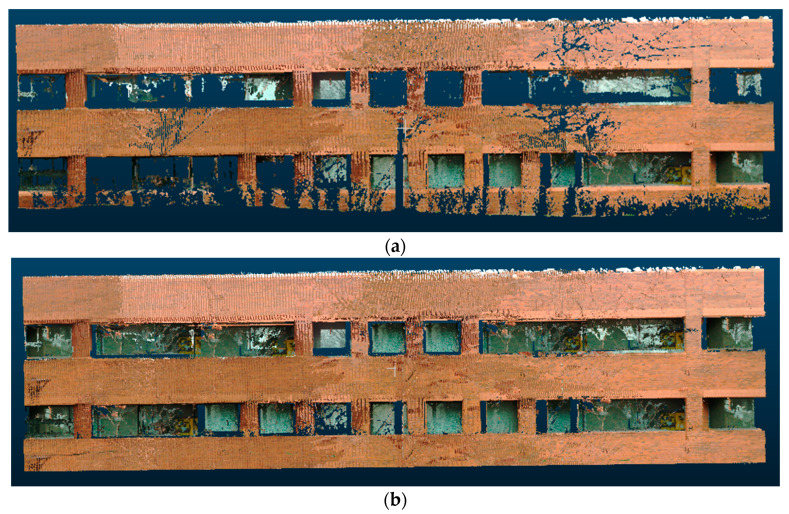
Visual comparison between (**a**) natural ground truth and (**b**) manual ground truth for the Pettit building scene.

Natural ground truth vs. manual ground truth: this study mainly used the technique of combining multiple scans to generate a “natural” ground truth for scene completion. However, this form of ground truth data still contains missing data due to limitations in scanning hardware and scanning time. To investigate how much this will bias the comparison results of point cloud completion, we made several samples of “manual” ground truth data by shifting and merging points from the existing ground truth point cloud to create a more complete point cloud. These manually modified point clouds thus consist of a combination of real and synthetic data. The point cloud completion results were then re-evaluated with the manual ground truth and compared to the natural ground truth data for the Pettit building scene (Figure 18). Results in Table 6 show that the evaluated metrics show generally worse results when using the “manual” ground truth since it could account for many points that were not included in the “natural” ground truth. However, the relative performance between different scene completions was still maintained.

Computation time analysis: Table 7 shows the computation time required for each method to perform scene completion. The computation time was recorded for each of the 11 scenes in the test data and averaged over all scenes. Results show that the proposed method had a somewhat longer computation time than some of the baseline methods, but it was still reasonable. The results also show that the method using partial convolutions took an especially long time since it required the manual step of creating image masks.

Comparison with point-based neural networks: this section provides a performance comparison with several end-to-end methods for point cloud completion that use point-based neural networks such as PCN [7], TopNet [54], and FoldingNet [55]. These methods have different architectures but are based on the same principle of directly predicting an output point cloud from an input point cloud using a neural network. In this study, PCN, TopNet, and FoldingNet were implemented using a forked repository from the original Completion3D benchmark [67]. For a fair comparison, each network was retrained with our building facade dataset using the same cross-validation scheme as described in Section 3.2.2. Similar to the proposed method, each network was trained with 467 point clouds and tested with 11 point clouds. The point clouds were also preprocessed using the same normalization procedure, which is to center at the origin and to rescale to unit length. Note that these methods did not directly predict the color information; thus, we used a nearest neighbor search to assign the color channels for the output point cloud.

**Figure 19:**
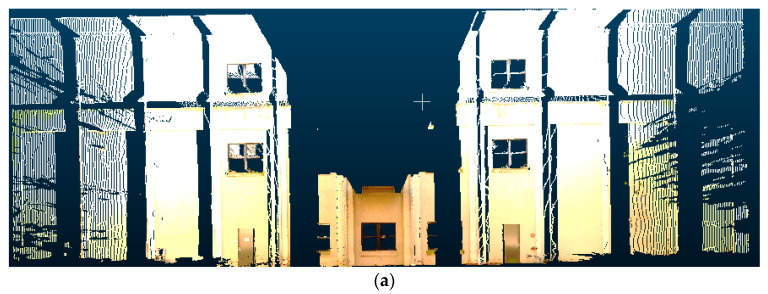

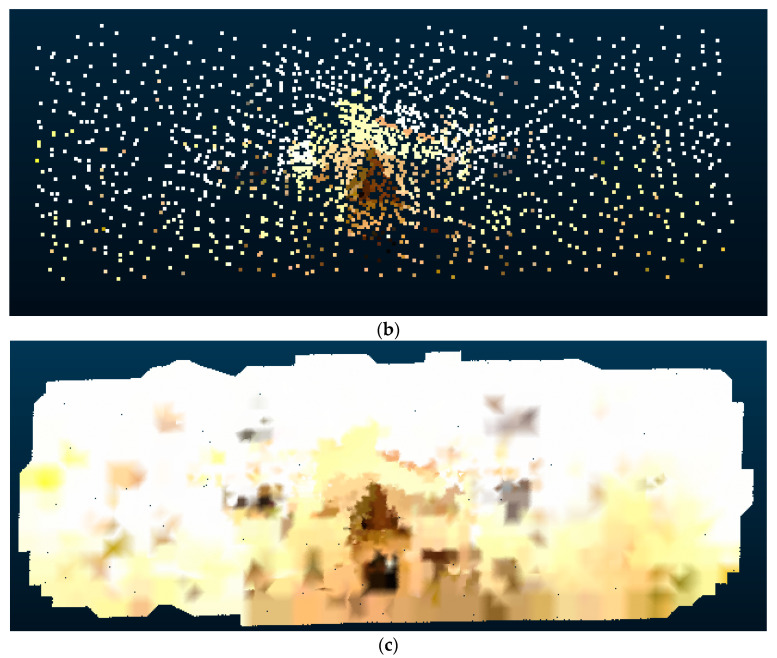
Point cloud scene completion results using point completion network (PCN): (**a**) input point cloud, (**b**) output point cloud, and (**c**) output point cloud after upsampling.

Figure 19b shows the output point cloud predicted by PCN given the input point cloud in Figure 19a. In the original implementation of PCN [7], the output point cloud was limited to 16,384 points and the point cloud was clearly much sparser compared to the input point cloud. This study used a mesh-based upsampling technique to increase the number of points to 1,000,000. However, results in Figure 19c show that the result still lacked the ability to represent detailed geometry due to the sparsity of the original prediction.

Table 8 shows a quantitative comparison of the scene completion performance metrics for PCN, TopNet, and FoldingNet, averaged across the test set of 11 building facades. The results show that the upsampled version had a higher recall rate across all three networks. However, there was still a significant gap between the performance of these point-based neural networks compared to the proposed method when applied to the building facade dataset. The main problem of these methods is that they only predict a constant number of output points, which could work well with point clouds of simple CAD models but not with large and complex building facades. Another problem is that these methods do not preserve the scale and offset information in the original point cloud and instead rely on an encoder–decoder structure to translate the input point cloud into an output point cloud.

Qualitative evaluation: Table 9 summarizes the qualitative improvement over other methods in the literature that were not directly evaluated. Compared to most existing methods in the literature, the proposed method is advantageous because it works directly with point cloud data, is able to reconstruct color information, and works for curved surfaces.

Limitations: there are several remaining limitations that need to be taken into account when using the proposed orthographic projection and generative adversarial inpainting method for point cloud scene completion. First, this study was mainly targeted towards scene completion of building facades to take advantage of the structure and symmetry in the scene. To apply this method for more general 3D scenes, a different set of training data needs to be used. Another limitation is the requirement to separately use orthographic projection to convert the 3D point cloud into a 2D image followed by depth remapping to convert the inpainted 2D image back into the 3D point cloud. To make the process more streamlined, the network architecture can be modified so that it is trainable end-to-end.

The targeted scenario for the application of the proposed scene completion method was mainly for the case of point cloud data collection with terrestrial laser scanning (TLS), which usually has the critical issue of occlusions and missing data. There are other alternatives to address the problem of occlusions such as using advanced laser scanners with multistation measurements, increasing the number of scan locations, or using advanced scan planning and optimization techniques. These scanning methods could still have missing data when taking multiple scans due to inaccessibility or occlusions that are too close to or in close contact with the target object (e.g., trees and shrubs). The main advantage of applying an automated scene completion algorithm is the ability to save manual labor and manual operation in laser scanning at the cost of having some uncertainty in the reconstructed point cloud. In any case, the authors expect that the idea of automated scene completion is still applicable to fix imperfections in the acquired data.

In this study, the point cloud preprocessing steps such as registration, segmentation, filtering, and extraction of individual facades from the original scene were carried out manually for simplicity and convenience. In an actual application, these steps can potentially be automated using established point cloud processing algorithms. For example, the point cloud scans can be automatically registered by feature point matching [68]. In addition, individual facades can be extracted using learning-based segmentation or recognition algorithms [69].

## 6. Conclusions

The objective of this research was to improve point cloud quality for 3D reconstruction, to improve visualization of point cloud scenes, to reduce scanning time for acquiring a complete point cloud scene, and to fix defective point cloud scans. To achieve this, we propose a method of performing point cloud scene completion of building facades using orthographic projection and generative adversarial inpainting. The proposed method was compared against several baseline methods on the task of reconstructing building facades in a dataset of laser-scanned point clouds. Through a comparison of several performance metrics based on both geometry and color, the proposed method is shown to achieve the best performance overall. In summary, the contributions of this research were as follows: (i) we proposed a framework necessary for 3D reconstruction of full building facades from incomplete scans using orthographic projection and 2D inpainting methods, (ii) we identified strengths and weaknesses of different scene completion methods through qualitative analyses, and (iii) we demonstrated the feasibility of scene completion for different scenarios including whole buildings and curved facades. For future work, the authors plan to further automate the scene completion pipeline by implementing an automatic segmentation algorithm for building facades before applying an orthographic projection and inpainting. The authors will also work on developing new methods for inpainting point clouds directly in 3D without having to perform orthographic projection. In addition, the authors will work on combining deep neural networks with other geometric methods (e.g., Poisson reconstruction, hole filling, or plane fitting) to improve the scene completion results.

## Figures and Tables

**Table 1:** Comparison of scene completion accuracy metrics (bold numbers indicate best performance).

Method	Voxel Precision (%)	Voxel Recall (%)	F1-Score (%)	Position RMSE (m)	Color RMSE
Poisson reconstruction	56.0	56.2	55.9	0.017	0.146
Hole-filling	77.3	54.8	63.3	**0.015**	**0.137**
Hybrid	48.7	54.9	51.1	0.018	0.152
Plane-fitting	14.0	47.3	21.0	0.018	0.189
Partial convolution	**87.7**	59.1	68.4	**0.015**	0.148
Proposed	78.1	**63.1**	**69.2**	**0.015**	0.145

**Table 2:** Accuracy comparison between different variations of the Pix2Pix network.

Method	Voxel Precision (%)	Voxel Recall (%)	F1-Score (%)	Position RMSE (m)	Color RMSE
Original	75.1	63.7	68.5	0.016	0.158
+ feature augmentation	76.4	63.8	69.0	0.016	0.151
+ feature augmentation + color smoothing	78.1	63.1	69.2	0.015	0.145

**Table 3:** Performance comparison between having the input image resized with the aspect ratio ignored vs. preserved.

Method	Voxel Precision (%)	Voxel Recall (%)	F1-Score (%)	Position RMSE (m)	Color RMSE
Ignore aspect ratio	87.5	68.8	76.0	0.015	0.141
Preserve aspect ratio	87.9	69.5	76.4	0.015	0.146

**Table 4:** Performance comparison between different neighborhood parameter settings for depth remapping.

Method	Voxel Precision (%)	Voxel Recall (%)	Color RMSE
1-Nearest Neighbor	87.5	68.8	0.141
2-Nearest Neighbor	82.4	63.2	0.174
10-Nearest Neighbor	82.2	62.9	0.174

**Table 5:** Performance comparison between using different voxel resolutions averaged over all eleven scenes.

Method	Voxel Precision (%)	Voxel Recall (%)	F1-Score (%)	Position RMSE (m)	Color RMSE
**Voxel Resolution of 0.01**					
Poisson reconstruction	15.2	5	6.4	0.0036	0.124
Hole-filling	43.2	41.3	42.8	0.0009	0.047
Hybrid	11.9	4.2	6.0	0.0037	0.133
Plane-fitting	6.0	4.0	11.9	0.0017	0.088
Partial convolution	49.6	41.8	43.3	0.0034	0.096
Proposed	58.4	40.2	46.5	0.0011	0.049
**Voxel Resolution of 0.05**					
Poisson reconstruction	56.0	56.2	55.9	0.017	0.146
Hole-filling	77.3	54.8	63.3	0.015	0.137
Hybrid	48.7	54.9	51.1	0.018	0.152
Plane-fitting	14.0	47.3	21.0	0.018	0.189
Partial convolution	87.7	59.1	68.4	0.015	0.148
Proposed	78.1	63.1	69.2	0.015	0.145
**Voxel Resolution of 0.2**					
Poisson reconstruction	69.7	72.1	70.5	0.069	0.172
Hole-filling	85.3	72.7	78.0	0.064	0.179
Hybrid	65.9	74.9	69.4	0.070	0.181
Plane-fitting	41.2	88.8	55.2	0.076	0.216
Partial convolution	91.9	78.2	77.6	0.064	0.178
Proposed	82.3	79.6	80.6	0.066	0.221

**Table 6:** Performance comparison between using natural ground truth and manual ground truth for the Pettit building scene.

Method	Voxel Precision (%)	Voxel Recall (%)	F1-Score (%)	Position RMSE (m)	Color RMSE
**Natural ground truth**					
Poisson reconstruction	52.9	58.7	55.6	0.017	0.105
Hole-filling	96.3	69.2	80.5	0.015	0.104
Hybrid	47.5	58.6	52.5	0.017	0.105
Plane-fitting	6.0	33.7	10.2	0.019	0.154
Partial convolution	98.4	69.5	81.5	0.015	0.104
Proposed	93.8	70.5	80.5	0.015	0.110
**Manual ground truth**					
Poisson reconstruction	57.5	40.4	47.5	0.018	0.125
Hole-filling	96.6	44.0	60.5	0.015	0.117
Hybrid	52.1	40.7	45.7	0.018	0.126
Plane-fitting	8.8	31.6	13.8	0.020	0.150
Partial convolution	98.6	44.2	61.0	0.015	0.117
Proposed	95.4	45.4	61.6	0.016	0.130

**Table 7:** Computation time comparison between different scene completion methods.

**Method**	**Computation Time (s)**	**Details**
Poisson reconstruction	106	
Hole-filling	26	-
Hybrid	184	-
Plane-fitting	13	-
Partial convolution	600	Includes time to perform orthographic projection and manual masking
Proposed	60	Includes time to perform orthographic projection

**Table 8:** Comparison of performance metrics with point-based neural networks.

Method	Voxel Precision (%)	Voxel Recall (%)	F1-Score (%)	Position RMSE (m)	Color RMSE
PCN	5.5	0.4	0.7	0.021	0.195
PCN (upsampled)	2.8	4.3	3.4	0.021	0.205
TopNet	4.9	0.2	0.4	0.020	0.255
TopNet (upsampled)	2.8	5.0	3.5	0.021	0.207
FoldingNet	3.6	0.2	0.4	0.021	0.196
FoldingNet (upsampled)	3.3	3.9	3.5	0.021	0.202
Proposed	78.1	63.1	69.2	0.015	0.145

**Table 9:** Qualitative comparison of the proposed scene completion method compared to other works in the literature.

Method	Reconstruct Color Information	Works Directly with Point Cloud Data	Works for Curved Surfaces	Can Reconstruct Arbitrary Classes of Objects
Pauly et al. [22]	No	Yes	Yes	Requires templates
ScanComplete [9]	No	No	Yes	Yes
SSCNet [10]	No	No	Yes	Yes
Adan et al. [13]	No	Yes	No	Restricted to walls, floors, ceilings
Proposed	Yes	Yes	Yes	Yes
